# Regulating root structure of potted lettuce to magnify absorption from APP and UAN fertilizers

**DOI:** 10.3389/fpls.2024.1407984

**Published:** 2024-05-31

**Authors:** Changqing Li, Yahao Li, Jungang Yang, Bingrui Lian, Jiqing Wang, Guoyuan Zou

**Affiliations:** ^1^ Institute of Plant Nutrition, Resources and Environment, Beijing Academy of Agriculture and Forestry Sciences, Beijing, China; ^2^ College of Resources and Environmental Sciences, Hebei Agricultural University, Baoding, China; ^3^ Department of Greenhouse Management, Beijing Cuihu Agricultural Technology Co., Ltd., Beijing, China; ^4^ College of Agriculture and Forestry Sciences, Hebei North University, Zhangjiakou, China

**Keywords:** urea ammonium nitrate solution (UAN), liquid ammonium polyphosphate (APP), lettuce, root architecture, nitrogen (N) and phosphorus (P) absorption

## Abstract

**Introduction:**

Improvement of root architecture is crucial to increasing nutrient acquisition.

**Methods:**

Two pot experiments were conducted to investigate the effects of different concentrations of urea ammonium nitrate solution (UAN) and ammonium polyphosphate (APP) on lettuce root architecture and the relationship between roots and nitrogen (N) and phosphorus (P) absorption.

**Results:**

The results showed that lettuce yield, quality, and root architecture were superior in the APP4 treatment compared to other P fertilizer treatments. The N480 treatment (480 mg N kg^-1^ UAN) significantly outperformed other N treatments in terms of root length, root surface area, and root volume. There were significant quantitative relationships between root architecture indices and crop uptake of N and P. The relationships between P uptake and root length and root surface area followed power functions. Crop N uptake was significantly linearly related to the length of fine roots with a diameter of <0.5 mm.

**Conclusion and discussion:**

The length of fine roots played a more prominent role in promoting N absorption, while overall root size was more important for P absorption. APP has a threshold of 9.3 mg P kg^-1^ for stimulating the root system. Above this threshold, a rapid increase in root absorption of P. UAN can promote extensive growth of fine roots with a diameter less than 0.5 mm. Applying appropriate rates of APP and limiting UAN application to less than 400 mg N kg^-1^ can improve root architecture to enhance N and P absorption by lettuce. These results highlight a new possibility to improve nutrients use efficiency while maintaining high yields.

## 1 Introduction

Lettuce is widely consumed as fresh-cut vegetables or side dishes in fast food, making it quite popular worldwide (FAOSTAT, 2020; Thapa et al., 2022). In recent years, lettuce cultivation in China has experienced rapid development, with an area approaching 10% of the national vegetable area (Jia et al., 2022). Especially with the widespread application of fertigation (drip irrigation and fertilization) management systems, lettuce cultivation in cold and arid regions of Northern China has enlarged rapidly and gradually formed large-scale production, contributing significantly to increased productivity and income for farmers in impoverished areas (Toonsiri et al., 2016; Tei et al., 2020; Shaik et al., 2022).

However, in lettuce cultivation, field management based on experience often lacks scientific and rational technical guidance, leading to excessive use of N and P fertilizers, low efficiency, and environmental pollution issues (Ying and Zhang, 2021). This not only disrupts the balance of soil microorganisms but also results in decreased vegetable yield and quality. Moreover, the expansion of cultivation areas and the high water consumption for irrigation pose significant challenges to local water resource security and increase the risk of ecological degradation, thereby affecting the sustainable development of the vegetable industry (Yuan et al., 2019; Yan et al., 2020; Ju et al., 2023). Therefore, it is urgent to improve the utilization efficiency of N and P fertilizers and to reduce the high consumption of irrigation water in lettuce cultivation, promoting the healthy development of soil and the industry.

Currently, conventional solid compound fertilizers are mainly used for fertigation in large-scale lettuce cultivation. However, conventional solid fertilizers dissolve slowly during the early cold spring, leading to uneven nutrient distribution and blockage of water pipeline, resulting in inconsistent growth of individual plants, reduced marketability, and decreased profitability for farmers (Rathore et al., 2021). High-quality water-soluble fertilizers are expensive, and most farmers tend to apply large amounts of low-cost fertilizers to achieve high yields. In China, the efficiency of N fertilizers is around 40% to 50%, with a loss rate of 50% to 60%, while the efficiency of P fertilizers is around 10% to 20%, with a loss rate of 80% to 90%, resulting in significant waste of N and P (Li et al., 2017; Jiang et al., 2021; Shen et al., 2022). Fertigation, which can significantly improve water and fertilizer utilization efficiency, has been most popular in vegetable cultivation (Kuscu et al., 2014). However, the dissolution and dispersion of solid fertilizers in drip irrigation systems are slowed down in cold and high-altitude regions where large-scale open-field vegetables are planted, resulting in blockage and lower efficiency of fertigation. Liquid fertilizers, containing one or more essential nutrients, have better flowability than solids at low temperatures. They disperse immediately when contacting with water. The use of liquid fertilizers can avoid the difficulties of solid fertilizer dissolution and the risk of clogging drip irrigation equipment. Moreover, they can be rapidly and uniformly injected into the drip irrigation system, thereby improving fertilization efficiency. Liquid APP is a solution of N and P elements, mainly composed of pyrophosphoric acid, with the addition of orthophosphoric acid and low-polymerized polyphosphates. APP can chelate trace elements in the soil and is a high-P concentration, low-pollution, and environmentally friendly liquid fertilizer (Mcbeath et al., 2006). Studies by Kuo et al. (2005) have shown that the polymeric P in polyphosphate ammonium weakens the fixation of P in the soil, thereby increasing the content of available P. Gao et al. (2019) found that the application of APP significantly improved the availability of P in the soil and mobilizes trace nutrients. UAN is a new type of liquid N fertilizer composed of urea, ammonium nitrate solution, and water, processed into a liquid fertilizer without a solid process. Maddux et al. (1991) conducted studies on N uptake and yield using N15 labeling techniques and found that applying UAN in banded application increased maize yield by 8.4% and N uptake by 13.3% while reducing soil residual nitrate-N by 26.6%. Grant (2014) observed the effects of different methods (UAN, urea, and urease inhibitor) on the grain yield of spring wheat through three-year field trials. They found that in the second and third years, the application of UAN without adding urease inhibitors resulted in higher spring wheat yields compared to urea broadcasting. Therefore, the use of new liquid fertilizers is an important way to reduce fertilizer input and increase nutrient use efficiency.

Reducing fertilizer inputs and promoting root absorption, regulating root architecture and nutrient supply to the root zone can provide technical support for mitigating excessive fertilizer use and losses of N and P through leaching (Lecompte et al., 2008; Shen et al., 2013; Wen et al., 2017). Bai et al. (2021) found that intercropping increased root volume and root surface area of wheat and faba bean under low P stress, thereby promoting P uptake. Li et al. (2022) demonstrated that tomato yield and N uptake in integrated management were significantly higher than those in a traditional practice by 32.1% and 39.7%, respectively, with an increase in the proportion of fine roots in the integrated management mode. Therefore, changes in root architecture are particularly important for improving crop yield and nutrient uptake. However, current researches on UAN solution and liquid APP primarily focus on their application, and there is limited information on their combined application and their effects on leafy vegetable growth, root morphology, and nutrient uptake efficiency. We hypothesized that appropriate concentrations of UAN and APP can optimize lettuce root architecture to promote the absorption of N and P. Here, two pot experiments were carried out. Our specific objectives were: (1) to investigate the effects of different concentrations of UAN solution, liquid APP, and their combination on lettuce root architecture, nutrient uptake, yield, and quality. (2) to evaluate the quantitative response of the root system and the potential for fertilizer reduction and efficiency improvement. Investigating these issues will provide information for improving N and P use efficiency together, and to find new possibilities of how to maintain yield when reducing the amount of fertilizers.

## 2 Materials and methods

### 2.1 Experimental site

**Figure 1:**
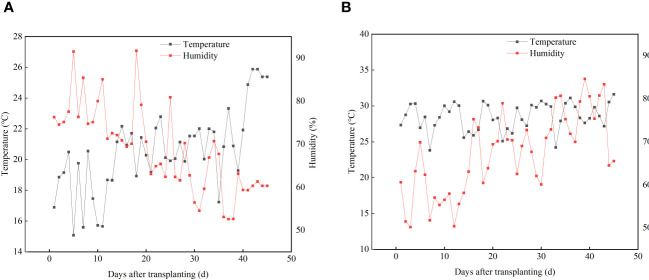
Temperature and humidity during the experiments of P **(A)** and N **(B)**.

Two experiments were conducted from March to July 2021 in a multi-span greenhouse at the Beijing Academy of Agricultural and Forestry Sciences (N39°56’36.6”, E116°17’9.6”). The greenhouse had glass walls on all sides, a translucent plastic roof, and was equipped with heating and cooling systems. Pot cultivation with peat substrate was adopted. The main properties of the substrate were: organic matter content of 45.0 g kg^-1^, pH of 6.0, total N content of 14.1 g kg^-1^, total P content of 1.80 g kg^-1^, total potassium content of 21.2 g kg^-1^, electrical conductivity of 240 μs cm^-1^, total porosity of 57.3%, and bulk density of 0.22 g cm^-3^. Experiments were carried out consecutively. The first experiment from March to April was about P; the second from May to July was about N. An automatic recorder was used to record the temperature and humidity in the experimental area. The average temperature and humidity during the P experiment were 19.76°C and 68.2%, and 22.8°C and 67.4% during the N experiment. The daily temperature and humidity are shown in **Figure 1**.

### 2.2 Experimental materials

Loose-leaf lettuce (cultivar: ‘Grand Rapids’) with a growth period of approximately 45 days was planted. The diameter of the pots is 25 cm, height is 18 cm, with 3.0 kg of substrate filled per pot. The substrate was primarily composed of peat, supplemented with vermiculite and perlite. The applied fertilizers included UAN (total N content 32%, containing 8% ammonium N, 8% nitrate N, and 16% urea N, produced by Luxi Chemical, China), APP (N 13%, P_2_O_5_ 37%, pale yellow liquid, produced by Wengfu Group, China), urea (N 46%, commercially available), and mono ammonium phosphate (MAP, N 10%, P_2_O_5_ 64%, industrial grade, white crystals, commercially available).

### 2.3 Experimental design

**Figure 2:**
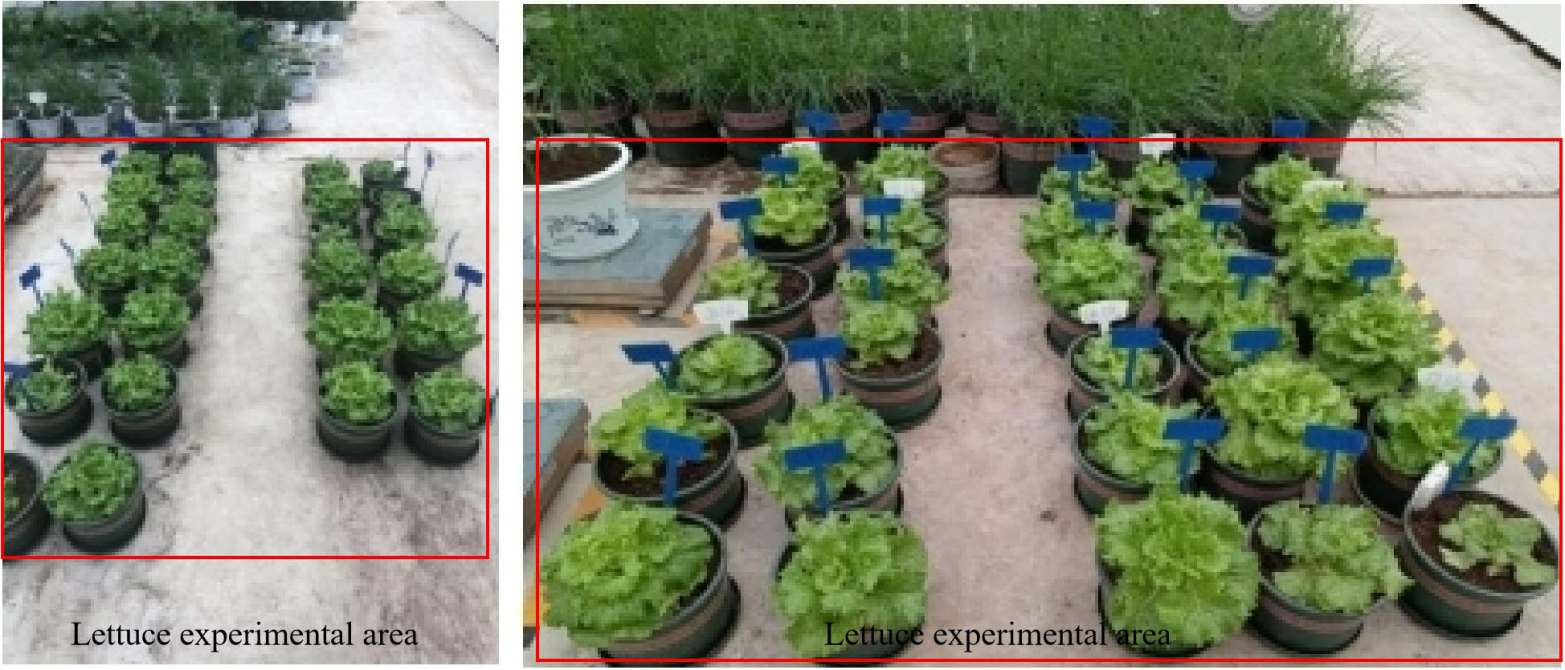
Pot experiments with APP and UAN in a multi-span glass greenhouse. (Left: P experiment, Right: N experiment).

This study adopted single factor test. Two pot experiments focused on N and P respectively. In the P experiment, five treatments were established, including four different concentrations of APP (0, 6.2, 9.3, 12.4 mg P kg^-1^) and one MAP treatment (15.5 mg P kg^-1^). For the N experiment, six treatments were: four different concentrations of UAN (0, 320, 400, 480 mg N kg^-1^), one UAN+APP combination treatment (400 mg N kg^-1^, 133 mg P kg^-1^), and one conventional urea treatment (480 mg N kg^-1^). The rates of APP and UAN for each treatment are shown in **Table 1**. Healthy seedlings were selected and one plant was planted per pot. A randomized block design with four replications for each treatment was adopted, totally 20 pots for the P experiment, and 24 pots for the N experiment (**Figure 2**). No basal fertilizer was applied in the P experiment, and all treatments received only top-dressing fertilization. The control group (CK) received an equal amount of water. Fifty percent of the total fertilizer amount was applied 10 days after transplantation, and the remaining 50% was applied on the 20th day, with the fertilizer dissolved in water. In the N experiment, both basal fertilizer and three additional top dressings were applied, accounting for 20%, 30%, 30%, and 20% of the total fertilizer amount, respectively. The top dressings were applied at 10, 20, and 30 days after transplantation. In the UAN+APP treatment, all the APP fertilizer was applied as basal fertilizer, while the UAN was applied following the same schedule as the other treatments. Except for APP and UAN, no other fertilizer was applied. Lettuce seedlings were transplanted when they reached the 3rd to 4th true leaf stage. The lettuce pots were rearranged weekly to ensure uniform growth conditions. Other managements such as plant protection, temperature, and humidity control were carried out according to the glass greenhouse practices.

**Table 1:** The amount of fertilizers applied for each treatment in P and N experiments.

Treatments	P (mg kg^-1^)	Treatments	N (mg kg^-1^)	P (mg kg^-1^)
CK	0	CK	0	–
APP2	6.2	N320	320	–
APP3	9.3	N400	400	–
APP4	12.4	N480	480	–
MAP	15.5	Urea	480	–
–	–	UAN+APP	400	133

### 2.4 Sample collection and measurement

Characteristics of the substrate: before the experiment, organic matter content (potassium dichromate-external heating method), total N (Kjeldahl method), total P (Molybdenum antimony anti-colorimetric method), total K (Flame photometry), pH (pH meter), total porosity (Drying method), and bulk density of the substrate used in the experiment (Ring knife method) were measured respectively. After the harvest, the residual available P (Molybdenum antimony anti-colorimetric method) and nitrate N content (Ultraviolet spectrophotometry) of substrates were tested (Bao, 2000).

Plant sampling and measurements: 45 days after the transplantation, lettuce plants were harvested by cutting at the base of the stem. The aboveground parts were collected, and the fresh weight of each plant was recorded. Then some samples were used to measure the content of vitamin C (2,6-dichloroindophenol method), soluble sugar content (Anthrone colorimetric method), and nitrate content (Ultraviolet spectrophotometry). The remaining samples were dried, and weighed to calculate the biomass, and then used to determine the total N content (Kjeldahl method) and total P content (Vanadium-molybdenum yellow colorimetric method) (Bao, 2000). The uptakes of N and P were calculated as the following formulas:

N uptake (g plant^-1^) = Total N content of the plant (%) × biomass (g plant^-1^)

P uptake (g plant^-1^) = Total P content of the plant (%) × biomass (g plant^-1^)

**Figure 3:**
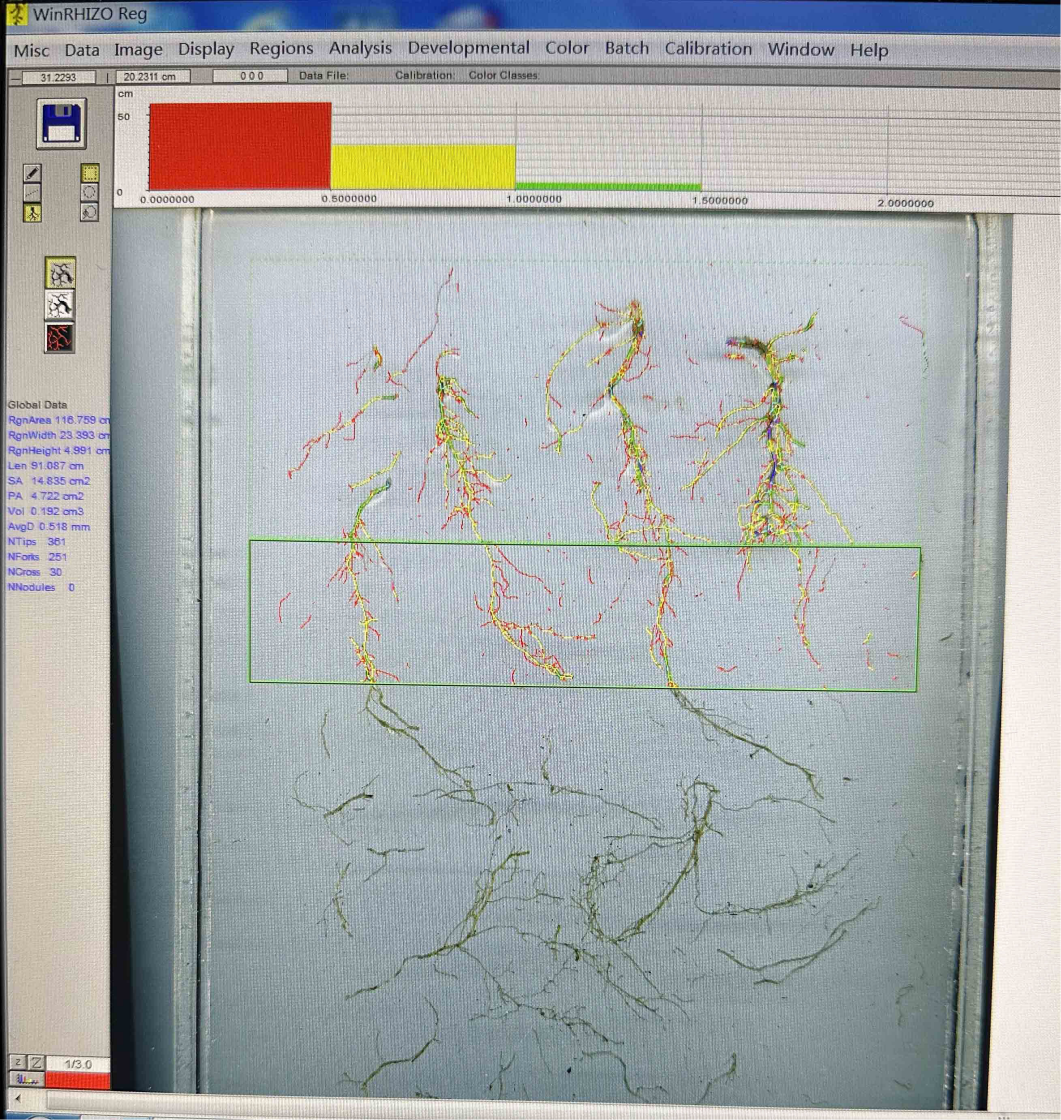
Root scanning image.

Root measurement: After harvesting the above-ground parts, the substrate was separated from the roots. The roots were soaked and washed with water to remove any substrate, and then carefully arranged into a scanning disk without overlapping. The roots were scanned to get an image. The WinRHIZO software (Regent, Canada) was used to analyze each image and obtain the root length, root diameter, root surface area, root volume, and root diameter distribution data (**Figure 3**).

### 2.5 Data analysis

Statistical analysis was performed by one-way ANOVA processes of SPSS 22 software (v22.0, Chicago, IL, USA). The means of each treatment were compared using the least significant difference (LSD) at a 0.05 level of significance for yield, N and P uptakes, and root architecture items. A Pearson correlation was used to calculate the correlation coefficients of root length, root surface area, root volume, and 0<D ≤ 0.5 with N uptake and P uptake. When a significant positive correlation between root items and P or N uptakes occurred, these indexes were fitted by regressive analysis. Power, linear, and polynomial function models were chosen to show the relationship between these data, in addition to a visual inspection of each type of response curve. The R^2^ values were used for model selection for each pair of data.

## 3 Results

### 3.1 Yield and nutrient uptakes

The application of UAN, APP, and the mixture of UAN and APP promoted the uptakes of N and P in lettuce, resulting in increased yield, with significant differences observed among the treatments (**Table 2**). The dry and fresh weight of lettuce increased with the increasing rate of APP, and the fresh weight of the APP4 treatment was significantly higher than those of other treatments. Compared to the MAP treatment, the fresh weight increased by 14.9% in the APP4 treatment, which reduced P input by 20%. The application of medium to high levels of UAN (N400 and N480) significantly increased the fresh weight of lettuce (**Table 2**). Compared to the urea treatment, the N480 treatment with an equal N rate showed a 17% increase in yield, while the N400 treatment with a 16.7% reduction in N input resulted in no significant yield reduction. When the N rate was reduced by 16.7% with APP added, the UAN+APP treatment showed a significant increase in yield. Overall, the yield of all treatments in the N experiment was lower than that in the P experiment, mainly due to the fact that lettuce is a cool-loving crop, and the increased temperature and humidity during the N experiment (**Figure 1B**) were unfavorable for lettuce growth and tending to infection by disease.

**Table 2:** Lettuce yield, N and P uptakes, and residual N and P content of substrate.

Treatment	Fresh weight (g plant^-1^)	Dry weight (g plant^-1^)	Plant N uptake (mg plant^-1^)	Plant P uptake (mg plant^-1^)	Residual P (mg kg^-1^)	Residual NO3^–^N (mg kg^-1^)
CK	67.59 ± 5.00 d§	7.08 ± 0.68 c	331 ± 61.6 d	13.5 ± 1.82 c	6.2 ± 1.05 b	–
APP2	79.80 ± 1.79 cd	8.68 ± 0.52 c	413 ± 78.6 d	10.2 ± 1.3 c	10.6 ± 3.34 a	–
APP3	93.13 ± 13.19 c	13.94 ± 2.46 b	628 ± 74.4 c	28.5 ± 3.51 b	11.5 ± 1.24 a	–
APP4	162.15 ± 10.92 a	22.63 ± 2.31 a	1133 ± 83.9 a	49.8 ± 7.01 a	9.0 ± 1.08 ab	–
MAP	141.14 ± 9.52 b	20.10 ± 0.69 a	890 ± 26.1 b	39.1 ± 6.68 ab	6.22 ± 0.87 b	–
CK	35.8 ± 0.86 c	5.84 ± 0.47 c	98.0 ± 9.44 e	-*	–	5.3 ± 0.10 d
N320	47.4 ± 2.25 c	7.95 ± 0.75 bc	137 ± 9.54 d	–	–	11.5 ± 0.88 bc
N400	64.9 ± 4.40 b	10.0 ± 1.00 a	166 ± 12.1 c	–	–	10.5 ± 0.40 abc
N480	70.1 ± 8.03 a	11.3 ± 0.16 a	211 ± 9.10 a	–	–	11.1 ± 1.03 ab
Urea	59.7 ± 3.87 b	10.2 ± 0.16 ab	179 ± 15.3 b	–	–	12.2 ± 0.68 a
UAN+APP	80.6 ± 4.14 a	13.2 ± 0.96 a	206 ± 17.9 a	–	–	9.18 ± 1.31 c

§Means followed by the same letter in the same list of each experiment do not differ using the LSD test (p ≤ 0.05), the same below. *Not measured.

The significant differences showed in N and P uptakes by lettuce (**Table 2**). In the P experiment, the APP4 treatment had the highest N and P uptake, significantly higher than the other treatments except for the MAP treatment. At the same time, the residual P content of APP4 (9.0 mg kg^-1^) was the lowest among all APP treatments at harvest time. In the N experiment, the N480 and UAN+APP treatments had significantly higher N uptakes than the other treatments. The UAN+APP treatment had a 16.7% reduction of N rate compared to the N480 and urea treatments while maintaining a high N uptake and low residual nitrate N content, which results in a significant improvement of N use efficiency. These results showed that reducing the dosage of APP or using it in combination with UAN solution can significantly promote lettuce’s yield and improve N and P uptake efficiency.

### 3.2 Lettuce’s leaf quality

Fertilization had a significant impact on the nitrate content, vitamin C (VC), and sugar content of lettuce (**Table 3**). In the P experiment, treatments with moderate to low rates of APP resulted in higher nitrate content in the leaves, whereas the lowest nitrate content occurred in the APP4 treatment, which decreased by 13.3% and 7.3% compared to the CK and MAP treatments, respectively. The application of APP significantly increased the vitamin C content, but there was no significant difference among the APP2, APP3, and APP4 treatments. The application of MAP did not promote an increase in VC content. Fertilizer application reduced the sugar content in lettuce, with no significant difference between the APP and MAP treatments.

**Table 3:** Effects of different concentrations of new liquid fertilizer on lettuce quality.

Treatment	Nitrate content (mg kg^-1^ FW)	Vitamin C content (mg 100g^-1^)	Soluble sugar content (%)
CK	970 ± 2.44 c	26.7 ± 2.09 c	2.48 ± 0.11 a
APP2	1733 ± 4.73 b	37.5 ± 1.44 a	2.18 ± 0.02 b
APP3	1782 ± 6.80 a	35.2 ± 1.44 ab	2.03 ± 0.07 bc
APP4	840 ± 2.26 e	33.7 ± 1.28 ab	1.93 ± 0.06 bc
MAP	906 ± 2.97 d	27.0± 0.83c	2.06 ± 0.14 bc
CK	944 ± 3.36 e	27.3 ± 1.35 d	2.36 ± 0.08 a
N320	1120 ± 22.9 d	29.6 ± 2.52 c	2.11 ± 0.18 ab
N400	1244 ± 10.5 c	31.4 ± 1.79 b	1.62 ± 0.11 c
N480	1412 ± 86.7 b	34.1 ± 2.15 a	2.05 ± 0.15 ab
Urea	1556 ± 11.5 a	29.9 ± 0.84 c	1.74 ± 0.14 bc
UAN+APP	1154 ± 20.9 d	36.2 ± 1.50 a	2.23 ± 0.04 a

In the N experiment, the nitrate content of leaves increased with increasing N rate. Under the equal N rate, the nitrate content in the N480 treatment was lower than that of the urea treatment. Among the fertilizer treatments, the UAN+APP treatment had the lowest nitrate content. Furthermore, the VC content and soluble sugar content in the UAN+APP treatment were significantly higher than in the other treatments, indicating a significant improvement in lettuce quality.

### 3.3 Root architecture

Except for the average root diameter in the N experiment, the application of UAN, APP, and their combination had a significant effect on the root length, surface area, root diameter, and root volume of lettuce (**Table 4**). In the P experiment, the root length and surface area increased with increasing P concentration in the three APP treatments, while the root diameter decreased with increasing P concentration, and the root volume remained unchanged. The APP4 treatment had the longest root length and the largest root surface area, and its surface area was significantly higher than the other treatments, increasing by 13.4% compared to the CK treatment. The APP2 and APP3 treatments had a significantly larger root diameter but lower root length compared to the other treatments. In the N experiment, the root length and surface area increased with increasing N rate in the three UAN treatments, and the N480 treatment showed significant increases in root length, surface area, and root volume compared with CK (**Table 4**). The root length, surface area, and root volume in the Urea and UAN+APP treatments were lower than in the N480 treatment, but there was no significant difference compared to the N400 treatment. It can be observed that the largest values of root length and surface area do not necessarily correlate with higher productivity and efficiency in the N experiment. Maintaining a balanced root system configuration, with an efficient match between root length and root diameter, is beneficial for nutrient uptake. The application of UAN in combination with APP (UAN+APP treatment) can form a root system configuration suitable for crop growth and nutrient absorption, resulting in higher yields while reducing N application by 17.6%.

**Table 4:** Effects of different concentrations of new liquid phosphate fertilizer on root architecture of lettuce.

Treatment	Root length (cm)	Root length density (cm cm^-3^)	Surface area (cm^2^)	Average diameter (mm)	Root volume (cm^3^)
CK	9286 ± 872 a	1.18 a	1624 ± 136 b	0.52 ± 0.02 c	27.8 ± 4.97 a
APP2	5174 ± 660 c	0.65 c	886 ± 73.9 d	0.66 ± 0.05 a	15.2 ± 2.99 b
APP3	6880 ± 1542 bc	0.88 bc	1123 ± 157 c	0.64 ± 0.07 ab	17.6 ± 3.67 b
APP4	9809 ± 1782 a	1.25 a	1841 ± 121 a	0.56 ± 0.07 c	16.0 ± 2.71 b
MAP	8784 ± 1633 ab	1.12 ab	1470 ± 136 b	0.56 ± 0.02 c	32.0 ± 4.86 a
CK	8105 ± 1133 c	1.04 c	1112 ± 150 c	0.48 ± 0.04 a	12.8 ± 1.37 c
N320	7999 ± 1297 c	1.02 c	1194 ± 203 bc	0.48 ± 0.02 a	15.1 ± 2.34 bc
N400	8884 ± 421 bc	1.13 bc	1364 ± 177 bc	0.47 ± 0.03 a	16.0 ± 0.87 ab
N480	12715 ± 1407 a	1.62 a	1724 ± 121 a	0.44 ± 0.03 a	18.7 ± 0.84 a
Urea	10033 ± 938 b	1.28 b	1431 ± 152 b	0.47 ± 0.03 a	16.4 ± 1.94 ab
UAN+APP	9890 ± 691 bc	1.26 bc	1498 ± 234 bc	0.42 ± 0.02 a	16.0 ± 2.06 ab

**Figure 4:**
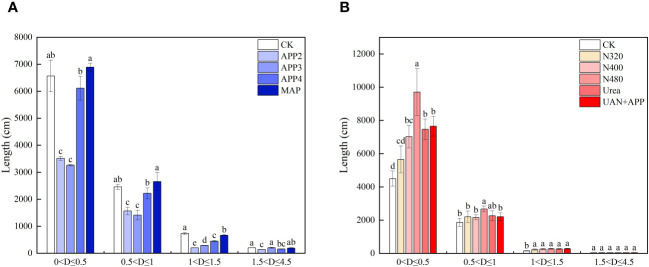
Effect of different concentrations of APP **(A)** and UAN **(B)** on root diameter classification (D-root diameter; Different lowercase letters above the columns indicate significant difference under p<0.05 level.).

The root system of each lettuce plant was divided into four sections based on diameter, from small to large (0<D ≤ 4.5 mm), and the root length was measured for each section (**Figure 4**). In the P experiment, the root system was mainly concentrated in the 0<D ≤ 0.5 mm and 0.5<D ≤ 1.0 mm diameter categories (**Figure 4A**). The CK and MAP treatments had the longest root length, which was significantly higher than that of the APP2, APP3, and APP3 treatments, with an increase of 7.43% to 111% (0<D ≤ 0.5 mm) and 10.7% to 88.2% (0.5<D ≤ 1.0 mm). For the three APP treatments, the APP4 treatment had a significantly higher root length than the APP2 and APP3 treatments. The same trend was observed in the 1<D ≤ 1.5 mm diameter category, and the differences between treatments gradually decreased as the root diameter exceeded 1.5 mm. In the N experiment, the root system was mainly distributed in the fine root section of 0<D ≤ 0.5 mm, accounting for 73.6% of the total root length. Except for the N480 treatment, which had a significantly higher proportion (97.5%) of roots smaller than 0.5 mm, there was no significant difference in the section of 0<D ≤ 0.5 mm between the N400, Urea, and UAN+APP treatments (**Figure 4B**).

### 3.4 Correlation analysis with root architecture

**Figure 5:**
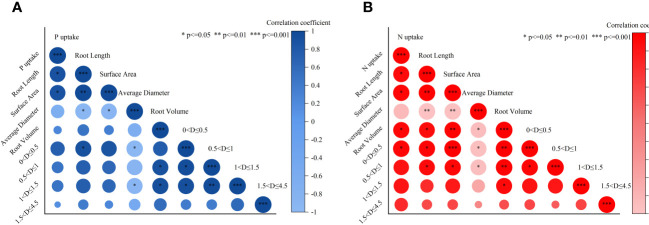
Correlation analysis between root architecture and P **(A)** and N **(B)** absorption of lettuce (The size of the circle represents the degree of correlation, the color from deep to shallow represents positive correlation to negative correlation, the asterisk in a circle represents significance between items.).

The correlation analysis between N and P uptakes and root characteristics in lettuce revealed significant relationships (**Figure 5**). Root length and root surface area exhibited a significant positive correlation with P uptake, indicating that an increase in root length and surface area was associated with higher P uptake. Similarly, root length, root surface area, root volume, and roots with a diameter of 0<D ≤ 0.5 mm showed a significant positive correlation with N uptake, suggesting that these root characteristics were positively associated with N uptake. Total root length and fine roots with a diameter ≤1.0 mm showed close correlations with other indicators, indicating their strong influence on root architecture and promotion of N and P uptake. Overall, N showed a more pronounced correlation with root characteristics compared to P, with increased correlation coefficients and significance, particularly for fine roots (0<D ≤ 0.5 mm) and N uptake.

**Figure 6:**
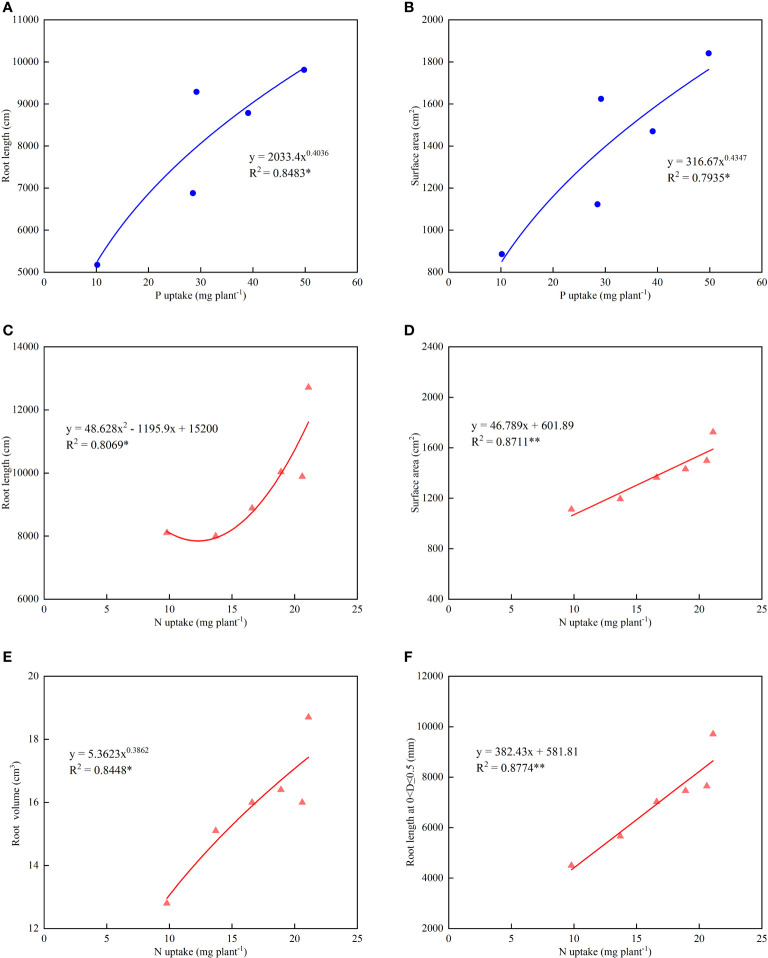
Relationship of root architecture and elemental absorption of lettuce **(A)** the relationship between root length and P uptake; **(B)** the relationship between root surface area and P uptake; **(C)** the relationship between root length and N uptake; **(D)** the relationship between root surface area and N uptake; **(E)** the relationship between root volume and N uptake; **(F)** the relationship between root at 0<D ≤ 0.5 and N uptake. (* indicates significance at the level of 0.05, ** indicates significance at the level of 0.01.).

Based on the significant positive correlations between P uptake and root length/surface area, as well as N uptake and root length, root surface area, root volume, and roots with a diameter of 0<D ≤ 0.5 mm, regression analysis was performed to determine the relationship between root characteristics and N/P uptake (**Figure 6**). The results showed that root length and root surface area exhibited a power function relationship with P uptake (y=2033.4x^0.4036^, R^2^=0.8483*; y=316.67x^0.4347^, R^2^=0.7935*). Root surface area and root length of roots with a diameter of 0<D ≤ 0.5 mm showed a linear relationship with N uptake (y=46.789x+601.89, R^2^=0.8711**; y=382.43x+581.81, R^2^=0.8774**). Root length demonstrated a quadratic function relationship with N uptake (y=48.628x^2^-1195.9x+15200, R^2^=0.8069), while root volume showed a power function relationship with N uptake (y=5.3623x^0.3862^, R^2^=0.8448). It can be observed that fine roots (0<D ≤ 0.5 mm) had the closest relationship with N uptake, exhibiting the highest determination coefficients and significance levels. On the other hand, total root length showed the closest relationship with P uptake, displaying the highest determination coefficient.

## 4 Discussion

### 4.1 Effects of APP and UAN on lettuce yield and quality

Fertigation has become an important technical measure in Chinese vegetable cultivation (Wang et al., 2021; Zheng et al., 2023), providing favorable conditions for the development of water-soluble fertilizers, especially liquid fertilizers. APP is composed of orthophosphates, pyrophosphates, and a portion of triphosphates and tetraphosphates, which have shown significant yield-increasing effects on crops (Chen, 2018). The results of this study showed that the dry and fresh weight of lettuce increased with increasing APP application, and the APP4 treatment exhibited significantly higher fresh weight compared to other treatments. In comparison to the MAP treatment, the APP4 treatment with 20% less P showed a 14.9% increase in fresh weight while reducing leaf nitrate content by 7.3% (**Tables 2**, **3**), which is consistent with the findings of Holloway et al. (2001). This can be attributed to the hydrolysis of APP into orthophosphates, which can be readily absorbed and utilized by plants. APP can provide a sustained nutrient supply for crop growth, prolonging the fertilization effect. Additionally, APP can easily combine with ions such as iron, aluminum, calcium, and magnesium in the soil, reducing the fixation of P and enhancing its mobility, thereby facilitating the movement of phosphate ions to the plant roots and improving P uptake (Torres-Dorante et al., 2005; Gao et al., 2019; Wang et al., 2019).

UAN is a liquid fertilizer that combines nitrate, ammonium, and amide forms of N. It releases N at a rate that combines the advantages of quick-acting and slow-release fertilizers, thereby improving crop yield and nutrient use efficiency. The results of this study showed that, compared to the Urea treatment, the N480 treatment (equivalent N level) and UAN+APP treatment (reduced N by 16.7% and additional APP) significantly increased lettuce yield by 17.4% and 35.0%, respectively. The UAN+APP treatment exhibited the lowest nitrate content in the leaves, and higher vitamin C (VC) and soluble sugar content compared to other treatments, which is consistent with the findings of Wang et al. (2018). Previous studies by Li et al. (2020) have shown that compared to the single application of N or P fertilizers, the combined application of N and P fertilizers resulted in the highest photosynthetic efficiency and improved yield. Cui et al. (2018) also found that the combined application of N and P fertilizers effectively increased nutrient uptake efficiency and crop yield. In conclusion, the application of liquid APP, UAN, and the mixture of UAN and APP at a reduced application rate can significantly promote lettuce yield and quality. Among them, the UAN+APP treatment demonstrated the best one, enhancing N and P uptake efficiency and achieving increased yield and improved quality.

### 4.2 Response of lettuce root architecture to APP and UAN and its implications for N and P uptake

A well-developed root architecture facilitates the absorption and utilization of water and nutrients by crops (Catherine and Bledsoe, 1999; Li et al., 2006; Liu et al., 2014; Jungers et al., 2019). Previous studies have indicated that root morphology is correlated with two forms of N, ammonium-N, and nitrate-N. Ammonium-N can be directly utilized by plant roots, stimulating the proliferation of fine roots (Gruber et al., 2013) and promoting root growth hormone accumulation (Meier et al., 2020). Nitrate-N, on the other hand, converting into ammonium within cells before being utilized by plants. It can also effectively promote the elongation growth of plant roots and stimulate the growth of fine roots (Yi et al., 2019). The results of this study showed that the root length and root surface area of the three UAN treatments increased with increasing N concentration. The N480 treatment exhibited significant increases in root length, root surface area, and root volume, with fine roots (less than 0.5 mm in diameter) accounting for 97.5% of the total root length, significantly higher than the Urea treatment at an equivalent N level, also significant yield and N absorption increase, and lower leaf nitrate content. This suggests that an appropriate concentration and ratio of ammonium and nitrate N can significantly increase the proportion of fine roots while improving production. Though the proportion of fine roots in the UAN+APP treatment was lower than that in the N480 treatment (**Figure 4B**), it had the highest yield and best quality. The residual nitrate N content of the substrate treated with UAN+APP at harvest time was significantly lower than that of other treatments, indicating that its aboveground absorption capacity was enhanced (**Table 2**). Hu et al. (2019) found that applying P fertilizer in combination with high N levels promoted rice growth and significantly increased biomass, which is consistent with the findings of this study. An appropriate N-P supply ratio can enhance the allocation of resources to the above-ground part, resulting in less resource allocation to the roots and an asynchronous growth relationship between the above-ground and below-ground parts (Luo et al., 2016). In the P experiment of this study, the root length and root surface area of the three APP treatments increased with increasing P concentration, with the APP4 treatment exhibiting the highest values, particularly in root surface area, which is consistent with the findings of Liang (2017). Under low P concentrations (APP2 and APP3 treatment), root growth was inhibited, and leaf nitrate content increased (**Figure 4A** and **Table 3**). Previous studies have shown that low P stress can inhibit the transport and assimilation of NO_3_^−^ in plants, thus affecting growth and biomass accumulation (Qi et al., 2010; Paredes et al., 2011). The results of this study show that there is a concentration threshold for the stimulatory effect of APP on root growth, that is root absorption increased rapidly when the supply concentration exceeded the threshold, 9.3 mg P kg^-1^ from the APP3 treatment. The possible reason for this phenomenon is that increased P concentration (APP4 treatment), more roots at 0<D ≤ 0.5 mm and 0.5<D ≤ 1.0 mm were generated (**Figure 4A**), leading to a significant increase in N and P uptake (**Table 2**). It was reported that intercropping has been shown to increase root volume and surface area, resulting in increased P uptake under low P supply during the seedling stage (Bai et al., 2021; Ren et al., 2021). Here the application of APP can also be magnified root absorption at a lower concentration above the threshold. The present study revealed significant quantitative correlations between root architecture indicators and N, and P uptake. With increasing root length and root surface area, P uptake followed a power function relationship (**Figures 6A**, **B**), while N uptake exhibited a significant linear relationship with fine roots (0<D ≤ 0.5 mm) (**Figure 6F**). Fine roots played a critical role in promoting N uptake, while overall root size was more crucial for P uptake. The application of appropriate concentrations of APP and reduced UAN dosage can improve root architecture and enhance N and P uptake efficiency.

In short, lettuce roots responded positively according to different forms, types, and concentrations of N and P fertilizers. The values of root length and root surface area are not simply the larger the better for achieving high yield and efficiency. Maintaining an appropriate root architecture that is well-matched to the environmental conditions, along with an efficient balance between root length and root diameter, is beneficial for nutrient absorption and continuously improving nutrient acquisition capacity, ultimately leading to higher crop yields (Li et al., 2022).

## 5 Conclusion

Optimizing root architecture are particularly important for improving crop yield and nutrient uptake. The length of fine roots played a more prominent role in promoting N absorption, while overall root size was more important for P absorption. APP has a threshold of 9.3 mg P kg^-1^ for stimulating the root system. Above this threshold, a rapid root absorption of P occurred. UAN can promote extensive growth of fine roots with a diameter less than 0.5 mm. Applying appropriate rates of APP and limiting UAN application to less than 400 mg N kg^-1^ can improve root architecture to enhance N and P absorption by lettuce. Therefore, forming a good root architecture through reasonable nutrient supply can expand the nutrient absorption of vegetables and ultimately improve the yield and quality. It would be a new possibility to improve nutrients use efficiency while maintaining high yields and quality with reduced rate of liquid UAN and APP fertilizers.

## Data availability statement

The original contributions presented in the study are included in the article/supplementary material. Further inquiries can be directed to the corresponding authors.

## Author contributions

CL: Data curation, Formal analysis, Investigation, Methodology, Software, Validation, Visualization, Writing – original draft. YL: Data curation, Formal analysis, Investigation, Methodology, Software, Validation, Visualization, Writing – original draft. JY: Funding acquisition, Project administration, Resources, Supervision, Writing – review & editing. BL: Investigation, Software, Writing – original draft. JW: Writing – review & editing. GZ: Conceptualization, Supervision, Visualization, Writing – review & editing.
